# Entropy in Exact 2D Navier–Stokes and 3D Burgers Gas Flows

**DOI:** 10.3390/e28020178

**Published:** 2026-02-03

**Authors:** Philip Broadbridge

**Affiliations:** Department of Mathematical and Physical Sciences, La Trobe University, Bundoora, VIC 3086, Australia; p.broadbridge@latrobe.edu.au

**Keywords:** compressible vortex, Navier–Stokes, vector Burgers’ equation, entropy, shock solution

## Abstract

Two exact solutions are constructed for viscous compressible gas dynamics in two and three dimensions. The first is a steady vortex, with explicit solutions for the full Navier–Stokes system of velocity, density, temperature and pressure. In contrast, the second is a time-dependent radial solution to the 3D vector Burgers’ equation, with a constant injection rate from a spherical interior surface. That solution is shock-like at low Reynolds numbers. In both cases, expressions are given for the local density of entropy production.

## 1. Introduction

Non-reactive fluid flows generate heat by viscous dissipation, as work is done against internal resistance to flow. This results in an increase in thermodynamic entropy. The relationship between entropy increase and dynamical instability is an active area of research [1,2,3]. One interesting coupling between heating and material dynamics occurs in viscous compressible gases, as both temperature and density affect pressure gradients. However, there are very few exact non-trivial compressible Newtonian fluid flows in three or even two dimensions. A weakly compressible time-dependent perturbation to an incompressible vortex has been constructed in the form of a similarity solution [4,5]. That solution assumes constant Prandtl number. However both viscosity and thermal conductivity are temperature dependent in real materials. Nihoul [6] gave examples with $\left(K,\mu \right)=\left(K_{0},e^{-\left(T-1\right)/T_{0}}\right)$ and $\left(K,\mu \right)=\left(\frac{1}{T},\frac{1}{T}\right)$, which has some relevance for incompressible liquids. For gases, we must allow for non-uniform density and for a viscosity that increases with temperature. The first aim here is to produce an exact solution for a steady two-dimensional circular flow in a compressible viscous fluid that has viscosity μ(T) increasing with temperature *T* in a physically meaningful way.

The vortex that is considered is a restricted type of flow, being steady and circular, with no radial velocity. Although the density of the fluid is constant following the circular motion, the density, temperature and fluid speed still vary in the radial direction. Therefore the second aim is to construct an explicit solution for time-dependent radial flow in three dimensions. Burgers [7], Cole [8] and Hopf [9] were interested in Burgers’ equation partly because of its resemblance to the Navier–Stokes momentum equation. It is still of interest to study its weak zero-viscosity limit, which is of the form of a Riemann shock in gas dynamics. In particular, it is of interest to calculate the entropy jump across the shock [10,11]. Because of that application, it is important to know that the vector Burgers’ equation, with or without a conservative external force, is also conditionally integrable, subject to the constraint of irrotationality. This fact is also widely known but not widely reported in the published literature (see e.g., Ref. [12]). The full class of vector equations that are linearised by the Hopf–Cole transformation, including the Madelung fluid formulation of quantum mechanics, is given in Ref. [13]. In the 1940s, Florin [14] had recognized the integrability of the potential Burgers’ equation in three space dimensions, for the purpose of modeling soil compaction. For this and other examples of conditionally integrable partial differential equations, the reader is referred to Ref. [15]. Knowledge of shock behavior has been gained largely from the one-dimensional Burgers’ equation. Tsai [16] studied the effect of a standard boundary condition on shock dynamics. Among other solutions, a two-dimensional radial cylindrical solution of the vector Burgers’ equation was constructed in Ref. [12]. In this article, a three-dimensional radial compressible flow solution will be constructed, with constant exit velocity from a fixed spherical surface. The Reynolds number may be varied at will, indicating shock behavior in the limit of zero viscosity.

The organization of the paper is as follows:

Section 2 sets up the Navier–Stokes system of equations for a steady vortex flow of a compressible Newtonian fluid. In fact it will be seen that the spatially dependent entropy source term can be expressed explicitly, along with explicit expressions for the Navier–Stokes system of density, velocity temperature and pressure in terms of familiar special functions or their integrals. After Section 2.1 outlines the general approach, Section 2.2 produces such an exact solution, in terms of easily programmable special functions, for all of those variables, plus an expression for the local entropy source for a temperature-dependent inert gas.

Section 3 recounts the general theory of the vector Burgers’ equation that remains integrable when constrained by the condition of irrotationality.

Section 4 constructs the exact three-dimensional radial solution of the vector Burgers’ equationBurgers’ equation, illustrating the development of shock at high Reynolds number. This time-dependent solution contrasts with the Newtonian fluid vortex of Section 2 that is incapable of producing a shock. The exact solution for radial velocity is effected by a sequence of Hopf–Cole and Carslaw–Jaeger transformations that transforms the radial Burgers’ equation in three dimensions to the one-dimensional linear diffusion equation.

Section 5 gives some conclusions, including some areas that will require future study.

## 2. Steady Vortex in 2D: Mass, Momentum, Heat and Entropy

The Navier–Stokes system for compressible fluid in *n*-dimensional Euclidean space (e.g., Ref. [17]), includes the coupled momentum, mass and heat transport equations. There can also be added, a relation for non-conserved thermodynamic entropy. Assuming the Einstein summation convention that the repeated indices are summed, in Cartesian coordinates the equations that account for steady momentum, mass and heat transport are

(1) $$\begin{array}{ccc} \rho u^{j}{𝜕}_{j}u_{i} & = & -{𝜕}_{i}P+{𝜕}_{j}\Sigma_{i}^{\;j}\;\;\;\;i=1,2,\cdots n, \end{array}$$

(2) $$\begin{array}{ccc} {𝜕}_{i}\left(\rho u^{i}\right) & = & 0, \end{array}$$

(3) $$\begin{array}{ccc} {𝜕}^{i}\left(K{𝜕}_{i}T\right)+\Gamma & = & 0. \end{array}$$

The dependent variables are density ρ, pressure *P*, fluid velocity $u^{j}$, and temperature *T*. The trace of the stress tensor is included in the isotropic pressure component $-P\delta_{i}^{\;j}$. The trace-free component is the deviatoric stress tensor $\Sigma_{i}^{\;j}$. Materials are modeled by imposing constitutive relations for the pressure *P* and for $\Sigma_{i}^{\;j}$. In a Newtonian fluid,

(4) $$\Sigma_{i}^{\;k}=2\mu \left[D_{i}^{\;k}-\frac{1}{3}\delta_{i}^{\;k}D_{j}^{\;j}\right]+\mu_{V}\;\delta_{i}^{\;k}D_{j}^{\;j},$$

where *K* is the thermal conductivity, μ is the shear viscosity and $\mu_{V}$ is the bulk viscosity, all of which are assumed to depend on temperature *T*. $D_{j}^{\;i}$ is the rate-of-strain tensor, or symmetric gradient of velocity, $D_{j}^{\;i}=\frac{1}{2}\left[{𝜕}_{j}u^{\;i}+{𝜕}^{i}u_{j}\right]$. Γ is the rate of energy dissipation per unit n-dimensional volume, due to the working of shear stresses against viscous resistance. That is, $\Gamma =\Sigma_{i}^{\;j}D_{j}^{\;i}$.

### 2.1. Circular Flow of a General Compressible Viscous Fluid

From the steady zero-divergence mass flow (2) in two dimensions, there is a mass-based stream function ψ(x) such that $\rho u=\nabla \times \psi e_{z}$. This form already satisfies the mass conservation equation. Hence for a circular flow about the origin,

(5) $$\rho u^{1}=\frac{x^{2}}{r}\psi^{\prime }\left(r\right),\;\;\;\rho u^{2}=-\frac{x^{1}}{r}\psi^{\prime }\left(r\right)\;\;\;\mathrm{and}\;\;u=Ue_{\theta };\;U=-\frac{\psi^{\prime }\left(r\right)}{\rho }.$$

Then the dissipative heat source simplifies to

(6) $$\Gamma =\mu r^{2}{\left[\omega^{\prime }\left(r\right)\right]}^{2};\;\;\;\mathrm{where}\;\;\omega =\frac{U}{r}.$$

Since u·∇ρ=0, it is true also that ∇·u=0. However, this does not mean that the density is uniform. For any radial function ρ(r) and circumferential u, the divergence of ρu is still zero as required. This means that the fluid need not be incompressible, even though the density following the circular flow is constant. There can be a radial variation of density and a radial variation of pressure that, in a steady circular flow, will drive the centripetal acceleration of the fluid. For a circular flow, $D_{j}^{\;j}=0$. Consequently the bulk viscosity has no effect on the dynamics.

The heat equation reduces to

(7) $$2K\left(T\right)\frac{T^{\prime }\left(r\right)}{r}+r\frac{d}{dr}\left(K\frac{T^{\prime }\left(r\right)}{r}\right)+\mu r^{2}{\left[\omega^{\prime }\left(r\right)\right]}^{2}=0.$$

From the radial and circumferential components of the momentum equation for Couette flow (e.g., [6]),

(8) $$-\rho \omega^{2}r+\frac{dP}{dr}=0,$$

and

(9) $$\begin{array}{c} \mu r^{2}\frac{d}{dr}\left\{\frac{1}{r}\frac{d\omega }{dr}\right\}+4\mu \frac{d\omega }{dr}+r\mu^{\prime }\left(T\right)T^{\prime }\left(r\right)\frac{d\omega }{dr}=0\\ \Leftrightarrow \frac{d}{dr}\log\left|\Lambda \right|=-\frac{d}{dr}\log\mu -\frac{4}{r}, \end{array}$$

where $\Lambda =\frac{1}{r}\frac{d\omega }{dr}$. Equation (9) integrates to $\Lambda =a_{1}r^{-4}\mu^{-1}$ with $a_{1}$ constant. This integrates further to

(10) $$\omega \left(=U/r\right)=a_{1}\int_{\infty }^{r}r_{1}^{-3}\mu^{-1}dr_{1}+a_{2},$$

with $a_{2}$ constant. Hence $a_{2}$ is the component of angular velocity due to rigid rotation, which as usual, will here be neglected when considering compressible fluids.

Now the heat Equation (7) takes the form

(11) $$2K\left(T\right)\frac{T^{\prime }\left(r\right)}{r}+r\frac{d}{dr}\left(K\frac{T^{\prime }\left(r\right)}{r}\right)+\frac{a_{1}^{2}\mu^{-1}\left(T\left(r\right)\right)}{r^{4}}=0.$$

Equivalently, with $\eta =\int_{\infty }^{T}K\left(T_{1}\right)dT_{1}<0$,

(12) $$\eta^{\prime \prime }\left(r\right)+\frac{1}{r}\eta^{\prime }\left(r\right)+\frac{a_{1}^{2}}{r^{4}}\mu^{-1}\left(T\left(\eta \right)\right)=0.$$

This can be solved exactly for various cases of μ(T) and K(T). From (8) and (10),

(13) $$\frac{𝜕P}{𝜕\rho }\rho^{\prime }\left(r\right)+\frac{𝜕P}{𝜕T}T^{\prime }\left(r\right)=\rho r{\left[a_{1}\int_{\infty }^{r}r_{1}^{-3}\mu^{-1}dr_{1}\right]}^{2}.$$

After assuming a given constitutive law for *P*, this may be regarded as an equation for density.

### 2.2. A Gas Whose Viscosity Increases with Temperature

Here, it will be assumed that

(14) $$\mu =c_{1}T^{1/2}$$

with $c_{1}$ as the constant. In fact, this is exactly the form given by classical kinetic theory for a dilute inert gas. Calculations in irreversible statistical thermodynamics involve approximate solutions of the Boltzmann equation. One standard approach is the Chapman–Enskog expansion [18]. Calculations for the hard-sphere gas are somewhat simpler than for other systems of interacting particles. From classical kinetic theory, μ is proportional to density and to mean free path *ℓ*. However, *ℓ* is inversely proportional to density, resulting in μ being a function of *T* alone. At low density, one commonly used expression has (14) with

(15) $$c_{1}=\frac{5}{16}\frac{1}{4\pi r_{0}^{2}}\sqrt{\pi m_{a}k_{B}},$$

where $m_{a}$ is the particle mass, $r_{0}$ is the particle radius, and $k_{B}$ is the Boltzmann constant. In fact, for general gases, μ(T) is often assumed to obey Sutherland’s law (e.g., Ref. [19], $\mu =c_{1}T^{3/2}/\left(T_{0}+T\right)$. This is close to (14) since $T_{0}$ is typically much less than room temperature.

**Figure 1:**
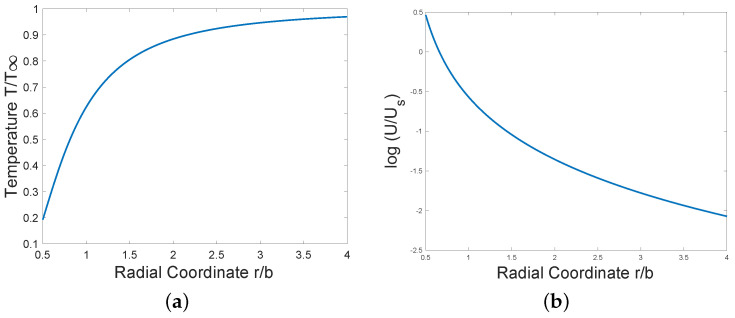
Exact compressible vortex solution for (**a**) temperature and (**b**) log (speed) of fluid.

Now (12) is a linear equation for η(r) if and only if $\mu^{-1}=c_{2}\eta +c_{5}$ with $c_{2}<0$ and $c_{5}>0$. This implies

$$\begin{array}{ccc} K & = & \frac{d\eta }{dT}\\ \Rightarrow K & = & \frac{1}{c_{2}}\frac{d}{dT}\mu^{-1}\\ & = & \frac{-1}{2c_{1}c_{2}}T^{-3/2}, \end{array}$$

which is positive and independent of $c_{5}$. That constant may be varied at will. For convenience it will be set to zero here. The general solution (Equation 2.1.2.132 of Ref. [20]) is written in terms of modified Bessel functions,

(16) $$\eta =c_{3}I_{0}\left(b/r\right)+c_{4}K_{0}\left(b/r\right),$$

where the interior length scale *b* is $\sqrt{-a_{1}^{2}c_{2}}$. Hereafter, a variable with subscript infinity will denote its limiting values as r→∞. Neglecting the second-kind modified Bessel function that diverges as r→∞, let $c_{4}=0$. The general solution that is bounded is equivalent to

(17) $$T=T_{\infty }I_{0}^{-2}\left(b/r\right),$$

the temperature at infinity being $T_{\infty }={\left(c_{1}c_{2}c_{3}\right)}^{-2}$, which can be freely prescribed. See Figure 1. Asymptotic values at large-*r* and small-*r* are given by $T/T_{\infty }=1-\frac{1}{2}{\left(\frac{b}{r}\right)}^{2}+O\left({\left[b/r\right]}^{4}\right)$ and $T/T_{\infty }∼2\pi \frac{b}{r}e^{-2b/r}$.

With μ now known explicitly as a function of *r*, circumferential speed U(r) and pressure P(r) can be obtained from (10) and (13) by direct integration. See Figure 1b. $U_{s}$ is a freely chosen constant of integration. Then

(18) $$\begin{array}{ccc} U & = & -a_{1}c_{2}c_{3}\;r\int_{r}^{\infty }r_{1}^{-3}I_{0}\left(b/r_{1}\right)dr_{1}\\ & = & U_{s}I_{1}\left(b/r\right), \end{array}$$

where the velocity scale is $U_{s}=-a_{1}c_{2}c_{3}/b$. At large-*r*, $U/U_{s}=b/2r+O\left({\left[b/r\right]}^{3}\right)$, agreeing asymptotically with the incompressible irrotational vortex. At small-*r*, $U/U_{s}∼e^{b/r}/\sqrt{2\pi b/r}$.

Now assuming the ideal gas law $\rho =\frac{m_{a}}{k_{B}}\frac{P}{T}$, where $m_{a}$ is the particle mass and $k_{B}$ is Boltzmann’s constant, (13) implies

(19) $$\begin{array}{c} P=P_{\infty }\exp\left\{\frac{-\rho_{\infty }T_{\infty }}{P_{\infty }}\int_{r}^{\infty }\frac{U^{2}\left(r_{1}\right)}{r_{1}T\left(r_{1}\right)}dr_{1}\right\}\\ \;\;\;\;\;\;\;\;\;\;\;\;\;=P_{\infty }\exp\left\{\frac{-\rho_{\infty }T_{\infty }}{P_{\infty }}\int_{0}^{q}\frac{U^{2}\left(q_{1}\right)}{q_{1}T\left(q_{1}\right)}dq_{1}\right\}\;\;\;\;\;(\mathrm{where}\;q=b/r),\\ \;\;\;\;=P_{\infty }\exp\left\{\frac{m_{a}}{k_{B}}c_{1}^{2}c_{2}^{3}c_{3}^{4}\int_{0}^{q}\frac{I_{0}^{2}\left(q_{1}\right)I_{1}^{2}\left(q_{1}\right)}{q_{1}}\;dq_{1}\right\}. \end{array}$$

$P_{\infty }$ is a free positive constant of integration. Other free parameters are $a_{1}$ and $c_{3}$ that enable $T_{\infty }$ and *b* to be freely specified. Then $\rho_{\infty }$ follows from the ideal gas law.

At large-*r*, $\log\left(P/P_{\infty }\right)=\frac{m_{a}}{k_{B}}c_{1}^{2}c_{2}^{3}c_{3}^{4}\frac{1}{8}{\left(b/r\right)}^{2}+O\left({\left[b/r\right]}^{4}\right).$ At small *r*, *P* rapidly approaches zero with logarithmic gradient of pressure

(20) $$\frac{d}{dr}\log\left(P/P_{\infty }\right)∼\frac{m_{a}}{k_{B}}\frac{c_{1}^{2}c_{2}^{3}c_{3}^{4}}{4\pi^{2}b}\frac{r}{b}e^{4b/r}.$$

**Figure 2:**
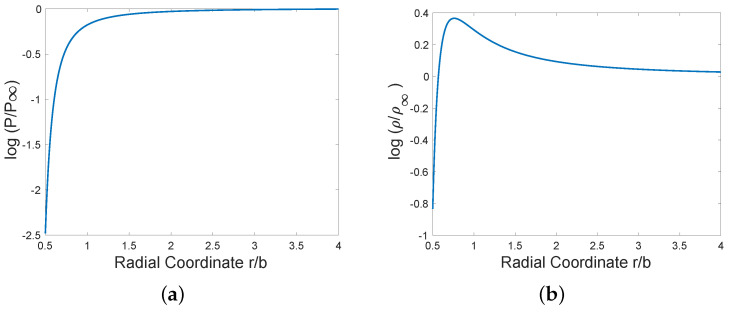
Exact compressible vortex solution for (**a**) log (pressure) and (**b**) log (density).

The integral within (19) has been evaluated by the trapezoidal rule with 800 equally spaced intervals for q∈[0.25,2], starting from the large−r approximate value of the integrand at q=0.25. For simplicity, in Figure 2 the value of the negative dimensionless parameter $c_{1}^{2}c_{2}^{3}c_{3}^{4}m_{a}/k_{B}$ is chosen to be −1. Note that at large−r, $\rho ∼1+\frac{1}{2}{\left(\frac{b}{r}\right)}^{2}.$ However, density decreases to zero as r→0 after achieving a maximum value where TdP/dr−PdT/dr=0. In the depicted example, that occurs at $\left(r,\rho \right)\approx \left(0.76b,1.44\rho_{\infty }\right)$.

The Clausius inequality has entropy increase bounded below by Γ/T. In accordance with that inequality, the entropy flux density can be regarded as the heat flux density divided by the temperature. Since u is normal to ∇T, the heat flux is solely due to nonlinear conduction, with no convection. Hence the equation for entropy density in a steady state with entropy production density $R^{\left(s\right)}$ is (e.g., [21])

(21) $$\begin{array}{ccc} 0=\frac{𝜕s}{𝜕t} & = & \nabla \cdot \left(\frac{K}{T}\nabla T\right)+R^{\left(s\right)} \end{array}$$

(22) $$\begin{array}{ccc} & = & \frac{1}{T}\nabla \cdot \left[K\nabla T\right]-\frac{K}{T^{2}}\nabla T\cdot \nabla T+R^{\left(s\right)} \end{array}$$

(23) $$\begin{array}{ccc} & = & -\frac{\Gamma }{T}-\frac{K}{T^{2}}{\left|\;\nabla T\;\right|}^{2}+R^{\left(s\right)}. \end{array}$$

Hence, $R^{\left(s\right)}=R_{1}^{\left(s\right)}+R_{2}^{\left(s\right)}$, where

(24) $$R_{1}^{\left(s\right)}=\frac{\Gamma }{T};\;\;\;R_{2}^{\left(s\right)}=\frac{K}{T^{2}}{\left|\;\nabla T\;\right|}^{2}.$$

From (6) and (10), $\Gamma =a_{1}^{2}r^{-4}\mu^{-1}$ with $\mu^{-1}=-c_{2}c_{3}I_{0}\left(b/r\right)$. From (16) and (17), the contribution to the entropy production rate due to the working of the stress is

(25) $$\frac{\Gamma }{T}=-a_{1}^{2}c_{2}c_{3}r^{-4}T_{\infty }^{-1}I_{0}^{3}\left(b/r\right).$$

The additional component, due to the occasional disruption of internal states with additional heat release as heat transfers by particle collisions, is

(26) $$\frac{K}{T^{2}}{\left|\;\nabla T\;\right|}^{2}=\frac{-2b^{2}}{c_{1}c_{2}}T_{\infty }^{-3/2}r^{-4}I_{0}\left(b/r\right)I_{1}^{2}\left(b/r\right).$$

Each of these contributions diminishes rapidly at large-*r*. The ratio of these two components is

(27) $$R_{2}^{\left(s\right)}/R_{1}^{\left(s\right)}=\frac{2b^{2}}{a_{1}^{2}c_{1}c_{2}^{2}c_{3}}T_{\infty }^{-1/2}I_{o}^{-2}\left(b/r\right)I_{1}^{2}\left(b/r\right).$$

This is of order ${\left(b/r\right)}^{2}$ at large-*r*, and of order 1 at small-r.

## 3. From Radial 3D Burgers to Uni-Dimensionalal Heat Equation

Exactly solvable Navier–Stokes systems are necessarily limited in their functional forms. The solution of the previous system is of a steady circular flow in two dimensions. By way of contrast, here a three-dimensional compressible flow will be constructed that is time dependent rather than steady, and radial rather than circumferential. However, it is a solution to the compressible vector Burgers’ equation rather than the Navier–Stokes system. The vector Burgers’ equation requires an implicit assumption that the kinematic viscosity μ/ρ is constant even though density is variable. Nevertheless, such evolving shock-like solutions of Burgers’ equation have been extensively studied, even in one dimension, because of the insight that they may provide on the morphology and dynamics of shocks in gas dynamics. In terms of Cartesian components, the vector Burgers’ equation is

(28) $${𝜕}_{t}u_{i}+u\cdot \nabla u_{i}-\nu \nabla^{2}u_{i}=0.$$

### Vector Hopf–Cole Transformation

By analogy with the Navier–Stokes system, u is usually regarded as a fluid velocity field. For irrotational flow, it is sufficient that

(29) $$u=-2\nu \frac{\nabla \Psi }{\Psi },$$

where Ψ(x,t) is any solution of the linear diffusion equation ${𝜕}_{t}\Psi =\nu \nabla^{2}\Psi$.

Now consider a radial solution of magnitude U(r,t), $u_{i}=U\left(r,t\right)\frac{x_{i}}{r}$, where *r* is the radial coordinate. As verified in Appendix A, it is sufficient that Ψ is a radial solution Ψ(r,t) of the linear heat equation

(30) $${𝜕}_{t}\Psi =\nu \Psi_{rr}+\frac{n-1}{r}\Psi_{r}.$$

Then $U=-2\nu \Psi_{r}/\Psi$. Some exact cylindrical radial solutions were constructed in [12]. The objective now is to construct a radial spherical solution. This is not a difficult task, given that in three dimensions, radial linear diffusion is equivalent to one-dimensional linear diffusion by the simple transformation (see Section 9.1 of the text by Carslaw and Jaeger [22])

(31) $$\Psi =\Phi /r\;\;;\;\;\Phi_{t}=\Phi_{rr}\;.$$

Impose the boundary condition for constant injection speed $u_{0}$ from a spherical surface r=a. Assume initial quiescence U=0 for r>a. It is natural to normalize to non-dimensional space the time and velocity variables r/a, $tu_{o}/a$ and $U/u_{0}$. Assume that variables have already been scaled in that way. Then, as is apparent from Appendix A, the boundary value problem to be solved is

(32) $$\begin{array}{c} U_{t}+UU_{r}=\bar{\nu }\left[U_{rr}+\frac{2}{r}U_{r}-\frac{2U}{r^{2}}\right], \end{array}$$

(33) $$\begin{array}{c} U(1,t)=1; \end{array}$$

(34) $$\begin{array}{c} U(r,t)\to 0,\;r\to \infty ; \end{array}$$

(35) $$\begin{array}{c} U(r,0)=0,\;\;r>1. \end{array}$$

where $\bar{\nu }$ is the reciprocal Reynolds number $\bar{\nu }=1/Re=\nu /au_{0}$. Since the rescaling of Ψ is a gauge transformation that leaves *U* invariant, there is no loss of generality in assuming Ψ(r,0)=1. Then the function Φ(r,t)=rΨ(r,t) satisfies Φ(r,0)=r for r>1. It is convenient to define χ=Φ−r, in terms of which the boundary value problem is

(36) $$\begin{array}{c} \chi_{t}=\bar{\nu }\chi_{rr}\;; \end{array}$$

(37) $$\begin{array}{c} \chi_{r}\left(1,t\right)+\left[\frac{1}{2\bar{\nu }}-1\right]\chi \left(1,t\right)=\frac{-1}{2\bar{\nu }}; \end{array}$$

(38) $$\begin{array}{c} \chi (r,t)\to 0,\;\;r\to \infty \;; \end{array}$$

(39) $$\begin{array}{c} \chi (r,0)=0. \end{array}$$

## 4. Exact Solution of Radial Velocity

Taking the Laplace transform $\chi \left(r,t\right)\to \tilde{\chi }\left(r,p\right)$,

(40) $$\begin{array}{c} p\tilde{\chi }={\tilde{\chi }}_{rr}\;; \end{array}$$

(41) $$\begin{array}{c} {\tilde{\chi }}_{r}\left(1,p\right)+\left[\frac{1}{2\bar{\nu }}-1\right]\tilde{\chi }\left(1,p\right)=\frac{-1}{2\bar{\nu }p}\;; \end{array}$$

(42) $$\begin{array}{c} \tilde{\chi }\left(r,p\right)\to 0,\;\;r\to \infty . \end{array}$$

The solution is

(43) $$\tilde{\chi }=\frac{\exp(-\left[r-1\right]\sqrt{p/\bar{\nu }})}{2p\sqrt{\bar{\nu }}}\frac{1}{\sqrt{p}-\sqrt{\bar{\nu }}\left[\left(1/2\bar{\nu }\right)-1\right]}.$$

**Figure 3:**
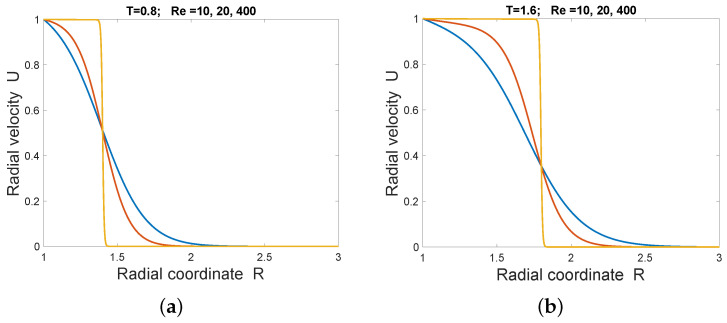
Exact solution U(r,t) progressively steepening at Re = 10 (blue), 20 (red), 400 (amber) at times (**a**) t = 0.8, (**b**) t = 1.6.

After inverting the Laplace transform (e.g., Ref. [23]), it follows that

(44) $$\begin{array}{c} \Psi =1+\frac{1}{r}\frac{1}{1-2\bar{\nu }}\left\{\exp\left(\begin{array}{c} -w \end{array}\left[r-1\right]+w^{2}\bar{\nu }t\right)erfc\left(\frac{r-1}{2\sqrt{\bar{\nu }t}}-w\sqrt{\bar{\nu }t}\right)-erfc\frac{r-1}{2\sqrt{\bar{\nu }t}}\right\}, \end{array}$$

where $w=\frac{1}{2\bar{\nu }}-1$. It follows that at fixed t and large *r*, the solution for U(r,t) is bounded above by $\exp(-\gamma r^{2})$, for some positive constant $\gamma <1/4\bar{\nu }t$. It also follows that at fixed r, U→1 as t→∞ (Figure 3).

An approximate fixed-form traveling shock front is apparent with speed approximately 0.5 when $\bar{\nu }\leq 1/400$. This follows theoretically from the fact that U(r,t) satisfies a pseudo-1D conservation equation $U_{t}+J_{r}=0$, where $J=\frac{1}{2}U^{2}-\bar{\nu }U_{r}-\bar{\nu }\frac{2}{r}U$. From the exact solution, at fixed *r*, U(r,t)→1 and $U_{r}\left(r,t\right)\to 0$ as t→∞. Hence $J\left(1,t\right)\to \frac{1}{2}-2\bar{\nu }$ as t→∞. The constructed solution has U(r,t)→0 and $U_{r}\left(r,t\right)\to 0$ as r→∞. If there is a shock wave at large *t* and small $\bar{\nu }$, by the Rankine–Hugoniot relation for the limiting Riemann shock wave for U(r,t), its speed must be

(45) $$\lim_{\bar{\nu }\to 0}\lim_{t\to \infty }\lim_{r\to \infty }\frac{J(1,t)-J(r,t)}{U(1,t)-U(r,t)}=\frac{1}{2}.$$

A radial traveling wave of constant speed can only occur in a compressible fluid that is becoming rarefied. Note, however, that the strong solutions with $\bar{\nu }>0$ cannot include an exact traveling wave. Reduction to a traveling wave follows from PDEs that are invariant under both time translations and space translations. The explicit *r*-dependent terms in the flux break that invariance. The value $\bar{\nu }=\frac{1}{2}$ (i.e., Re=2) is a special case, for which the solution is

(46) $$\Psi =1+\frac{1}{r}\left[\sqrt{\frac{2t}{\pi }}e^{-{\left(r-1\right)}^{2}/2t}-\left(r-1\right)erfc\frac{r-1}{\sqrt{2t}}\right].$$

For a radial compressible viscous flow, the rate of dissipative heating per unit volume takes the form

$$\begin{array}{ccc} \Gamma & = & 2\mu \left[{\left(\frac{𝜕U}{𝜕r}\right)}^{2}+2{\left(\frac{U}{r}\right)}^{2}-\frac{1}{3}{\left(\nabla \cdot u\right)}^{2}\right]\\ & = & 2\mu \left[U_{r}^{\;2}+2{\left(\frac{U}{r}\right)}^{2}-\frac{1}{3}{\left(U_{r}+\frac{2}{r}U\right)}^{2}\right]\\ & = & \frac{4}{3}\rho \nu {\left[U_{r}-\frac{U}{r}\right]}^{2}. \end{array}$$

This assumes negligible bulk viscosity, which is the case, for example, in monotomic gases. Hence the rate of dissipative heating per unit mass due to working against viscous stress, is $\frac{4}{3}\nu {\left[U_{r}-\frac{U}{r}\right]}^{2}.$

## 5. Conclusions

A steady axisymmetric circular flow of a compressible Newtonian fluid has been constructed here. The full Navier–Stokes system has been solved for velocity, density, temperature, and pressure in terms of integrals of powers of modified Bessel functions. This allows one to construct an explicit expression for the local entropy production rate. The solution has been worked through in detail when the viscosity has the same temperature dependence as for the Boltzmann hard-sphere monatomic gas. However, to allow a transformation of the nonlinear heat equation to a linear equation, thermal conductivity was taken to be a decreasing function of temperature. Nevertheless, the general approach indicates that solutions could be constructed for fluids with other constitutive laws. It remains to be investigated which of these can be more realistic. The solution has the property that density reaches a maximum value at some small radial distance from the center, where it approaches zero. The question remains as to whether the solution is unstable to a perturbation with vertical velocity component.

Circular two-dimensional steady flow is a strong limitation. Because of that, a time-dependent radial solution of the three-dimensional vector Burgers’ equation has also been constructed here. The solution is shock-like at low Reynolds numbers. Burgers’ equation has often been studied in connection with the thermodynamics of shocks. An expression for the local rate of entropy increase per unit mass has been given here. For the 3D radial flow, there is an apparent shock wave, but it is not exactly a traveling wave except in the inviscid limit when the wave speed has value 0.5 as in the unidimensional wave. From Figure 1, it can be conjectured that, as in the well-known one-dimensional traveling wave, the moving inflection point at the shock again occurs at a velocity value that is independent of viscosity, but unlike the unidimensional wave, the value of fluid speed at that point depends on time.

## Data Availability

The raw data supporting the conclusions of this article will be made available by the author on request.

## Appendix A. Evolution of Magnitude of Vector Field

In terms of Cartesian coordinates $x_{i}$, the components of radial unit vector ${\hat{e}}_{r}$ are $\frac{x_{i}}{r}$ where $r={\left(\sum_{i=1}^{n}x_{i}^{2}\right)}^{0.5}$. The components of radial flow velocity u are $u_{i}=U\left(r,t\right)\frac{x_{i}}{r}$.

$$\begin{array}{ccc} \nabla^{2}\left(U\frac{x_{i}}{r}\right) & = & \sum_{j=1}^{n}{𝜕}_{j}{𝜕}_{j}\left[U\frac{x_{i}}{r}\right]\\ & = & \sum_{j=1}^{n}{𝜕}_{j}\left[U_{r}x_{j}x_{i}r^{-2}-Ux_{i}x_{j}r^{-3}+\delta_{ij}Ur^{-1}\right]\\ & = & U_{rr}\frac{x_{i}}{r}+n\frac{U_{r}}{r}\frac{x_{i}}{r}+\frac{U_{r}}{r}\frac{x_{i}}{r}-\frac{U_{r}}{r}\frac{x_{i}}{r}\\ & \; & -2\frac{U_{r}}{r}\frac{x_{i}}{r}+3\frac{U}{r^{2}}\frac{x_{i}}{r}-\frac{U}{r^{2}}\frac{x_{i}}{r}-n\frac{U}{r^{2}}\frac{x_{i}}{r}\\ & \; & +\frac{U_{r}}{r}\frac{x_{i}}{r}-Ur^{-2}\frac{x_{i}}{r}\\ & = & \left[U_{rr}+\left(n-1\right)\frac{U_{r}}{r}+\left(1-n\right)\frac{U}{r^{2}}\right]\frac{x_{i}}{r}\\ & = & {𝜕}_{r}\left[r^{1-n}{𝜕}_{r}\left(r^{n-1}U\right)\right]\frac{x_{i}}{r}. \end{array}$$

Hence, the vector Burgers’ equation, ${𝜕}_{t}u_{i}+u\cdot \nabla u_{i}-\nu \nabla^{2}u_{i}=0$, reduces to

(A1) $$U_{t}+J_{r}=0;\;\;\;J=\frac{U^{2}}{2}-\nu r^{1-n}{𝜕}_{r}\left(r^{n-1}U\right).$$

That is,

(A2) $$U_{t}+UU_{r}=\nu U_{rr}+\nu {𝜕}_{r}\left[\frac{n-1}{r}U\right].$$

Equation (A2) for the magnitude of a radial vector satisfying the vector Burgers’ equation is not the same as the scalar Burgers’ equation except when n=1. However, it takes the form of a scalar conservation Equation (A1) in one dimension, with spatially dependent coefficients. With n=3, for Equation (A2) to be satisfied, it is sufficient that

(A3) $$U=-2\nu \Psi_{r}\left(r,t\right)/\Psi \;\;\mathrm{with}\;\;\Psi_{t}=\nu \nabla^{2}\Psi =\nu \left[\Psi_{rr}+\frac{n-1}{r}\Psi_{r}\right].$$

For any solution Ψ(r,t) of that linear scalar equation, the Hopf–Cole transformation gives a solution of Equation (A2) for the magnitude of a radial Burgers field. Equation (A2) is one of an integrable family of scalar equations [13],

(A4) $$U_{t}+UU_{r}=\bar{\nu }U_{rr}+{𝜕}_{r}\left[b_{1}\left(r\right)U\right]+b_{2}\left(r\right).$$

Substituting (A3) into (A2), one obtains

$$\begin{array}{c} \frac{-2\nu^{2}}{\Psi }\left[\Psi_{rrr}-\frac{n-1}{r^{2}}\Psi_{r}+\frac{n-1}{r}\Psi_{rr}\right]\\ +2\nu^{2}\frac{\Psi_{r}}{\Psi^{2}}\left[\Psi_{rr}+\frac{n-1}{r}\Psi_{r}\right]+4\nu^{2}\frac{\Psi_{r}\Psi_{rr}}{\Psi^{2}}-4\nu^{2}\frac{\Psi_{r}^{3}}{\Psi^{3}}\\ =\frac{\left(n-1\right)\nu^{2}}{r}\left[-2\frac{\Psi_{rr}}{\Psi }+2\frac{\Psi_{r}^{2}}{\Psi^{2}}\right]+\nu^{2}\left[-2\frac{\Psi_{rrr}}{\Psi }+2\frac{\Psi_{rr}\Psi_{r}}{\Psi^{2}}+4\frac{\Psi_{rr}\Psi_{r}}{\Psi^{2}}-4\frac{\Psi_{r}^{3}}{\Psi^{3}}\right]\\ +2\nu^{2}\frac{n-1}{r^{2}}\frac{\Psi_{r}}{\Psi }, \end{array}$$

which is identically true.
